# The genome sequence of the Mullein moth, *Shargacucullia verbasci *(Linnaeus, 1758)

**DOI:** 10.12688/wellcomeopenres.19757.1

**Published:** 2023-08-17

**Authors:** Mara K.N. Lawniczak, Peter W. H. Holland, Darwin Tree of Life Barcoding collective, Wellcome Sanger Institute Tree of Life programme, Wellcome Sanger Institute Scientific Operations: DNA Pipelines collective, Tree of Life Core Informatics collective, Darwin Tree of Life Consortium

**Affiliations:** 1 Wellcome Sanger Institute, Hinxton, England, UK; 2 University of Oxford, Oxford, England, UK

**Keywords:** Shargacucullia verbasci, Mullein moth, genome sequence, chromosomal, Lepidoptera

## Abstract

We present a genome assembly from an individual female *Shargacucullia verbasci* (the Mullein moth; Arthropoda; Insecta; Lepidoptera; Noctuidae). The genome sequence is 422.7 megabases in span. Most of the assembly is scaffolded into 32 chromosomal pseudomolecules, including the W and Z sex chromosomes. The mitochondrial genome has also been assembled and is 15.32 kilobases in length.

## Species taxonomy

Eukaryota; Metazoa; Eumetazoa; Bilateria; Protostomia; Ecdysozoa; Panarthropoda; Arthropoda; Mandibulata; Pancrustacea; Hexapoda; Insecta; Dicondylia; Pterygota; Neoptera; Endopterygota; Amphiesmenoptera; Lepidoptera; Glossata; Neolepidoptera; Heteroneura; Ditrysia; Obtectomera; Noctuoidea; Noctuidae; Cuculliinae; *Shargacucullia verbasci*; *Shargacucullia verbasci* (Linnaeus, 1758) (NCBI:txid987469).

## Background

The Mullein moth, *Shargacucullia verbasci*, is a member of the family Noctuidae with a wide geographic distribution across the northern Palaearctic. The moth has been recorded from Denmark and Sweden in the north to Portugal, Spain, Italy and Greece in the south; there are scattered records further east from Russia and Tajikistan ( GBIF Secretariat, 2022). In Britain, the species is most frequent in the south of England and across Wales, with no verified records from Scotland and Northern Ireland ( GBIF Secretariat, 2022; NBN Atlas Partnership, 2021). The moth was recorded in Ireland until 1952 before being declared locally extinct; it was rediscovered in 2021 ( Parnell, 2021).

**Figure 1.**
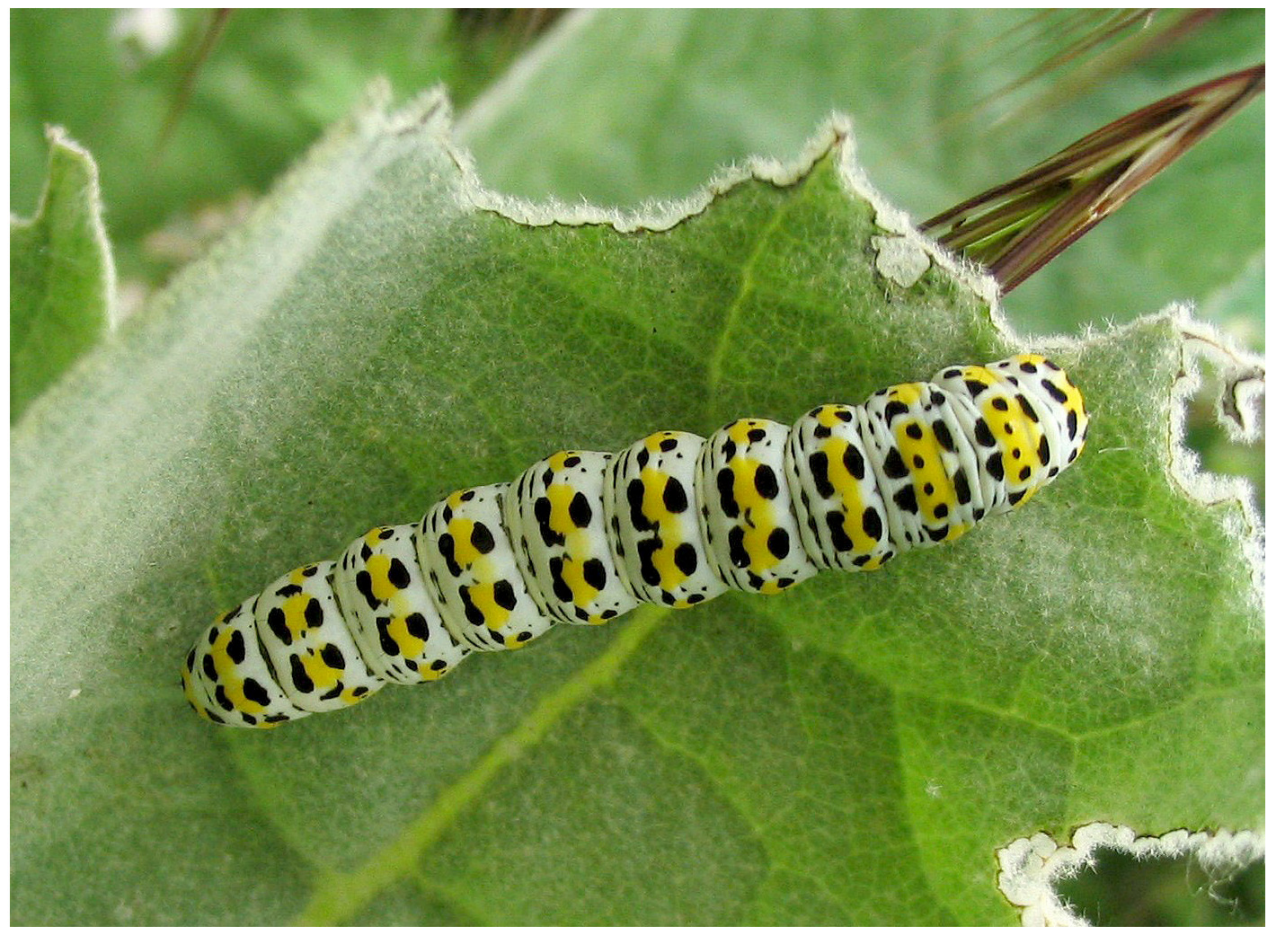
Photograph of *Shargacucullia verbasci* larva. Photographed near Bingen, Germany on 2005-06-05 by Hagen Graebner.

The adult moth is variable in size (wingspan 44–56 mm) with narrow pointed cream-coloured forewings edged in rich chocolate brown. The larval stage is more frequently encountered due to its diurnal feeding habit and conspicuous colouration, comprising white ground colouration with bands of bright yellow overlain with a regular pattern of black spots ( Figure 1). The usual larval food plants is mullein ( *Verbascum* sp.) with small numbers of larvae capable of stripping a plant completely of leaves; feeding has also been reported on figwort ( *Scrophularia* sp.) and Buddleia ( Bretherton *et al.*, 1983). The species has been proposed as a potential biological control agent for invasive *Verbascum* in North America ( Maw, 1980; Plant Conservation Alliance, 2005). In southern England, the adult moth is on the wing in April and May with the resultant larvae feeding through June and July; pupation occurs in a tough cocoon in the soil, with the pupal stage lasting up to 4 or 5 years ( Brooks, 1991).

A complete genome sequence for *Shargacucullia verbasci* will facilitate research into the evolution of food plant specificity in Lepidoptera and the molecular basis of pupal diapause. The genome of *S.verbasci*, based on one specimen from Saffron Walden, UK, was sequenced as part of the Darwin Tree of Life Project, a collaborative effort to sequence all named eukaryotic species in the Atlantic Archipelago of Britain and Ireland.

## Genome sequence report

The genome was sequenced from one *Shargacucullia verbasci* larva collected from Saffron Walden, UK (52.02, 0.25). The specimen was determined to be female based on its karyotype post sequencing. A total of 41-fold coverage in Pacific Biosciences single-molecule HiFi long was generated. Primary assembly contigs were scaffolded with chromosome conformation Hi-C data. Manual assembly curation corrected 14 missing joins or misjoins and removed 5 haplotypic duplications, reducing the scaffold number by 19.05%.

**Figure 2.**
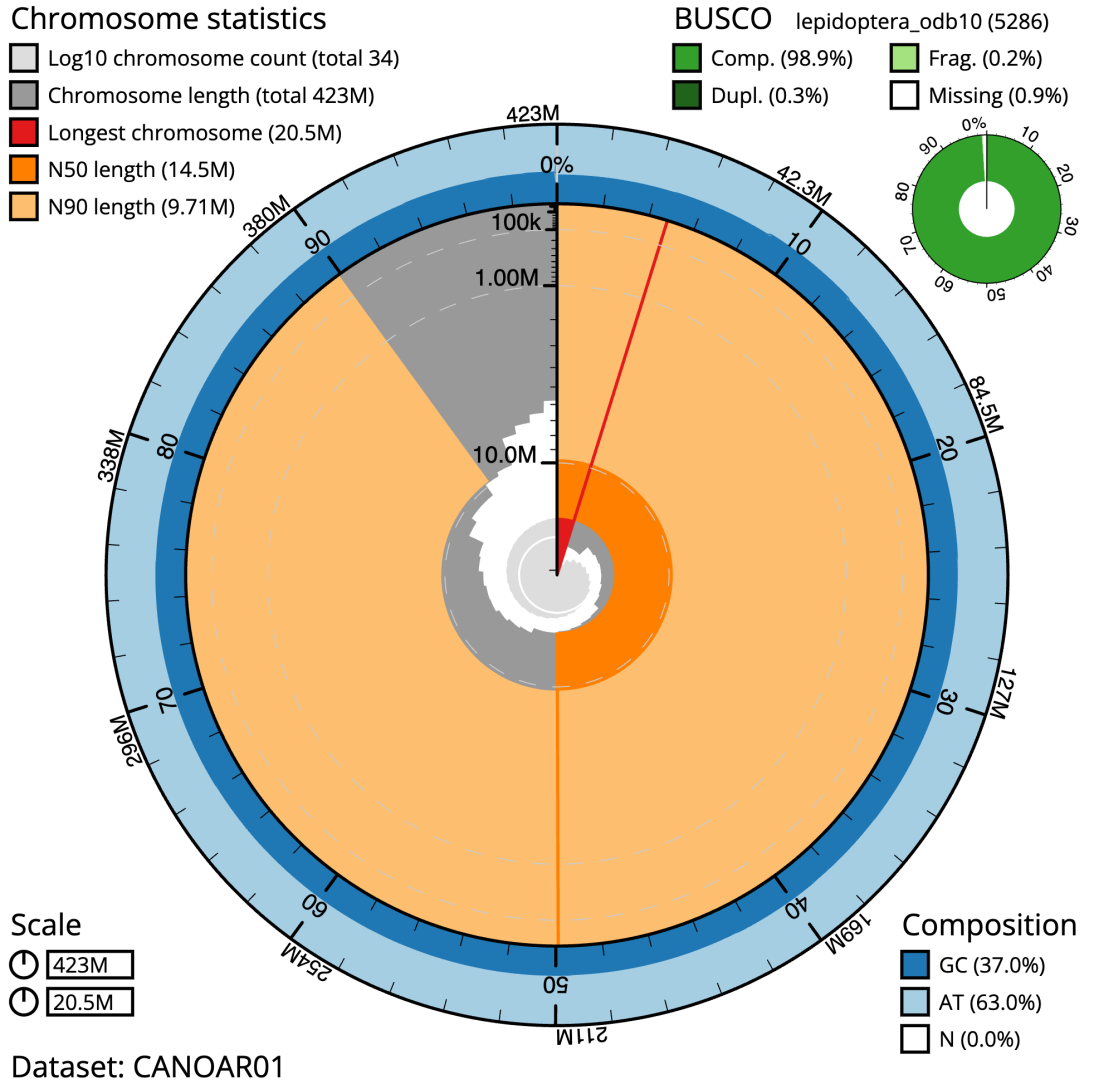
Genome assembly of *Shargacucullia verbasci*, ilShaVerb5.1: metrics. The BlobToolKit Snailplot shows N50 metrics and BUSCO gene completeness. The main plot is divided into 1,000 size-ordered bins around the circumference with each bin representing 0.1% of the 422,739,721 bp assembly. The distribution of scaffold lengths is shown in dark grey with the plot radius scaled to the longest scaffold present in the assembly (20,510,064 bp, shown in red). Orange and pale-orange arcs show the N50 and N90 scaffold lengths (14,523,181 and 9,713,589 bp), respectively. The pale grey spiral shows the cumulative scaffold count on a log scale with white scale lines showing successive orders of magnitude. The blue and pale-blue area around the outside of the plot shows the distribution of GC, AT and N percentages in the same bins as the inner plot. A summary of complete, fragmented, duplicated and missing BUSCO genes in the lepidoptera_odb10 set is shown in the top right. An interactive version of this figure is available at https://blobtoolkit.genomehubs.org/view/Shargacucullia%20verbasci/dataset/CANOAR01/snail.

The final assembly has a total length of 422.7 Mb in 33 sequence scaffolds with a scaffold N50 of 14.5 Mb ( Table 1). Most (99.98%) of the assembly sequence was assigned to 32 chromosomal-level scaffolds, representing 30 autosomes and the W and Z sex chromosomes. Chromosome-scale scaffolds confirmed by the Hi-C data are named in order of size ( Figure 2– Figure 5; Table 2). While not fully phased, the assembly deposited is of one haplotype. Contigs corresponding to the second haplotype have also been deposited. The mitochondrial genome was also assembled and can be found as a contig within the multifasta file of the genome submission.

**Table 1.** Genome data for *Shargacucullia verbasci*, ilShaVerb5.1.

Project accession data		
Assembly identifier	ilShaVerb5.1	
Species	*Shargacucullia verbasci*	
Specimen	ilShaVerb5	
NCBI taxonomy ID	987469	
BioProject	PRJEB56253	
BioSample ID	SAMEA7524383	
Isolate information	ilShaVerb5, female (larva): whole organism (DNA sequencing and Hi-C scaffolding)	
Assembly metrics *		*Benchmark*
Consensus quality (QV)	68.6	*≥ 50*
*k*-mer completeness	100%	*≥ 95%*
BUSCO **	C:98.9%[S:98.7%,D:0.3%],F:0.2%,M:0.9%,n:5,286	*C ≥ 95%*
Percentage of assembly mapped to chromosomes	99.98%	*≥ 95%*
Sex chromosomes	W and Z chromosome	*localised homologous pairs*
Organelles	Mitochondrial genome assembled	*complete single alleles*
Raw data accessions		
PacificBiosciences SEQUEL II	ERR10355970	
Hi-C Illumina	ERR10297870	
PolyA RNA-Seq Illumina	ERR10297869	
Genome assembly		
Assembly accession	GCA_947562105.1	
*Accession of alternate haplotype*	GCA_947562055.1	
Span (Mb)	422.7	
Number of contigs	82	
Contig N50 length (Mb)	9.5	
Number of scaffolds	33	
Scaffold N50 length (Mb)	14.5	
Longest scaffold (Mb)	20.5	

* Assembly metric benchmarks are adapted from column VGP-2020 of “Table 1: Proposed standards and metrics for defining genome assembly quality” from ( Rhie *et al.*, 2021). ** BUSCO scores based on the lepidoptera_odb10 BUSCO set using v5.3.2. C = complete [S = single copy, D = duplicated], F = fragmented, M = missing, n = number of orthologues in comparison. A full set of BUSCO scores is available at https://blobtoolkit.genomehubs.org/view/Shargacucullia%20verbasci/dataset/CANOAR01/busco.

**Figure 3.**
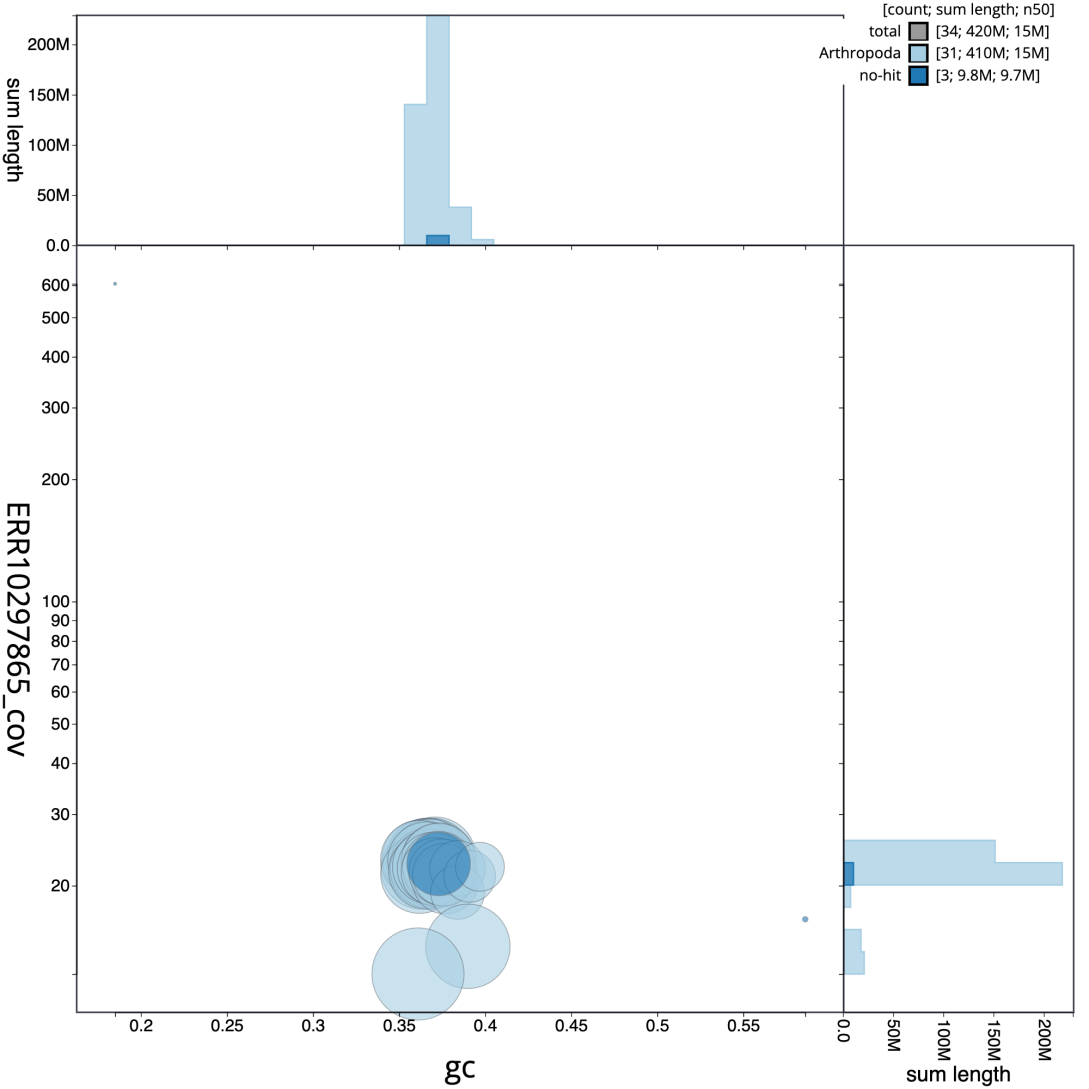
Genome assembly of *Shargacucullia verbasci*, ilShaVerb5.1: BlobToolKit GC-coverage plot. Scaffolds are coloured by phylum. Circles are sized in proportion to scaffold length. Histograms show the distribution of scaffold length sum along each axis. An interactive version of this figure is available at https://blobtoolkit.genomehubs.org/view/Shargacucullia%20verbasci/dataset/CANOAR01/blob.

**Figure 4.**
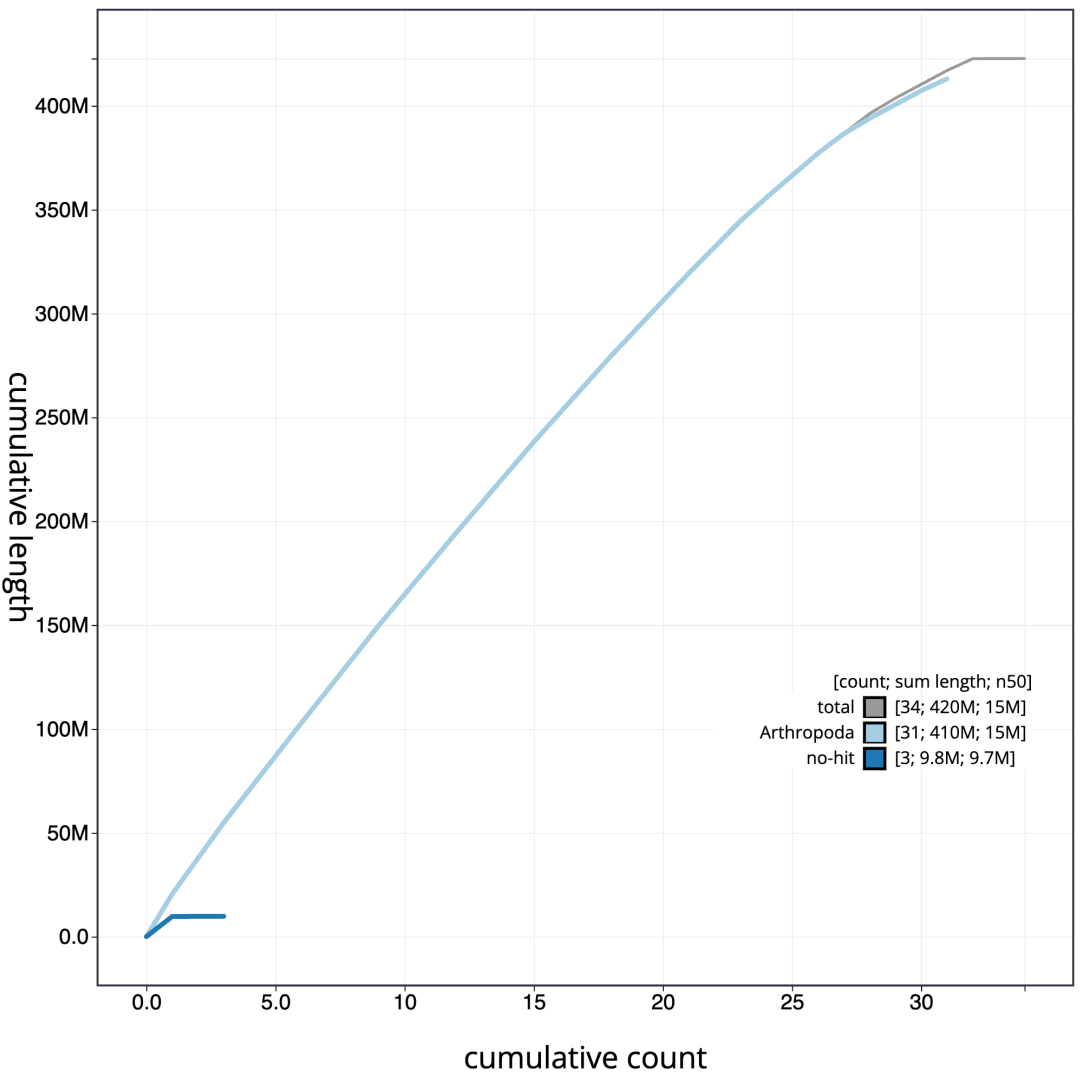
Genome assembly of *Shargacucullia verbasci*, ilShaVerb5.1: BlobToolKit cumulative sequence plot. The grey line shows cumulative length for all scaffolds. Coloured lines show cumulative lengths of scaffolds assigned to each phylum using the buscogenes taxrule. An interactive version of this figure is available at https://blobtoolkit.genomehubs.org/view/Shargacucullia%20verbasci/dataset/CANOAR01/cumulative.

**Figure 5.**
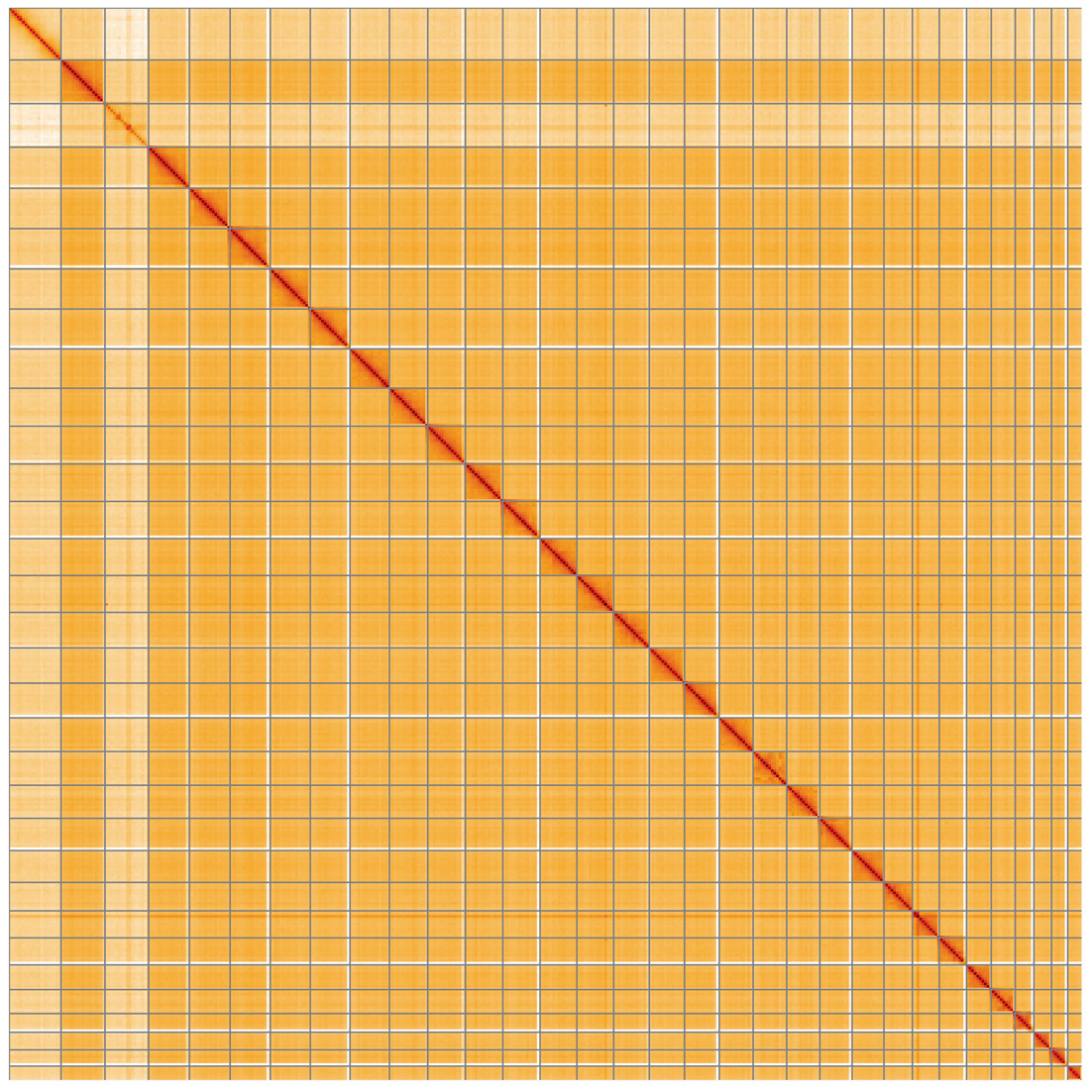
Genome assembly of *Shargacucullia verbasci*, ilShaVerb5.1: Hi-C contact map of the ilShaVerb5.1 assembly, visualised using HiGlass. Chromosomes are shown in order of size from left to right and top to bottom. An interactive version of this figure may be viewed at https://genome-note-higlass.tol.sanger.ac.uk/l/?d=fQ4fdfAvQrWrYfcAej1e_Q.

**Table 2.** Chromosomal pseudomolecules in the genome assembly of *Shargacucullia verbasci*, ilShaVerb5.

INSDC accession	Chromosome	Length (Mb)	GC%
OX387280.1	1	17.22	36.5
OX387282.1	2	16.1	37.0
OX387283.1	3	15.95	37.0
OX387284.1	4	15.84	37.0
OX387285.1	5	15.81	36.5
OX387286.1	6	15.67	36.5
OX387287.1	7	15.54	36.5
OX387288.1	8	14.97	36.5
OX387289.1	9	14.88	37.0
OX387290.1	10	14.75	36.0
OX387291.1	11	14.65	36.0
OX387292.1	12	14.52	36.5
OX387293.1	13	14.52	36.5
OX387294.1	14	14.03	36.5
OX387295.1	15	13.87	36.5
OX387296.1	16	13.61	37.0
OX387297.1	17	13.32	37.5
OX387298.1	18	13.28	37.5
OX387299.1	19	13.08	37.5
OX387300.1	20	12.67	37.0
OX387301.1	21	12.52	37.5
OX387302.1	22	11.18	37.0
OX387304.1	24	10.68	37.5
OX387303.1	23	10.68	37.5
OX387305.1	25	9.71	37.5
OX387306.1	26	9.42	37.5
OX387307.1	27	7.44	38.5
OX387308.1	28	6.91	38.5
OX387309.1	29	6.41	39.0
OX387310.1	30	5.71	39.5
OX387281.1	W	17.21	39.0
OX387279.1	Z	20.51	36.0
OX387311.1	MT	0.02	19.0

The estimated Quality Value (QV) of the final assembly is 68.6 with *k*-mer completeness of 100%, and the assembly has a BUSCO v5.3.2 completeness of 98.9% (single = 98.7%, duplicated = 0.3%), using the lepidoptera_odb10 reference set ( *n* = 5,286).

Metadata for specimens, spectral estimates, sequencing runs, contaminants and pre-curation assembly statistics can be found at https://links.tol.sanger.ac.uk/species/987469.

## Methods

### Sample acquisition and nucleic acid extraction

A female *Shargacucullia verbasci* (specimen ID SAN0001261, ToLID ilShaVerb5) larva was collected from Saffron Walden, UK (latitude 52.02, longitude 0.25) on 2020-06-02. The specimen was taken from a *Verbascum* plant in a garden by Mara Lawniczak (Wellcome Sanger Institute). This specimen was collected during the Covid19 lockdown and processed in a makeshift laboratory in ML’s bathroom. Using a scalpel to remove the head and forceps to dissect the specimen, the gut and its contents were removed to prevent excessive food plant material from being sequenced. The remainder of the caterpillar was processed into lentil sized pieces using a scalpel on a petri dish sitting on dry ice. The specimen was identified by Liam Crowley (University of Oxford) and preserved on dry ice.

The specimen was prepared for DNA extraction at the Tree of Life laboratory, Wellcome Sanger Institute (WSI). The ilShaVerb5 sample was weighed on dry ice with tissue set aside for Hi-C sequencing. Tissue of the whole organism was cryogenically disrupted to a fine powder using a Covaris cryoPREP Automated Dry Pulveriser, receiving multiple impacts. DNA was extracted at the WSI Scientific Operations core using the Qiagen MagAttract HMW DNA kit, according to the manufacturer’s instructions.

RNA was extracted from tissue from the whole organism of ilShaVerb2 in the Tree of Life Laboratory at the WSI using TRIzol, according to the manufacturer’s instructions. RNA was then eluted in 50 μl RNAse-free water and its concentration assessed using a Nanodrop spectrophotometer and Qubit Fluorometer using the Qubit RNA Broad-Range (BR) Assay kit. Analysis of the integrity of the RNA was done using Agilent RNA 6000 Pico Kit and Eukaryotic Total RNA assay.

### Sequencing

Pacific Biosciences HiFi circular consensus DNA sequencing libraries were constructed according to the manufacturers’ instructions. Poly(A) RNA-Seq libraries were constructed using the NEB Ultra II RNA Library Prep kit. DNA and RNA sequencing was performed by the Scientific Operations core at the WSI on Pacific Biosciences SEQUEL II (HiFi) and Illumina HiSeq 4000 (RNA-Seq) instruments. Hi-C data were also generated from tissue of ilShaVerb5 using the Arima2 kit and sequenced on the Illumina NovaSeq 6000 instrument.

### Genome assembly, curation and evaluation

Assembly was carried out with Hifiasm ( Cheng *et al.*, 2021) and haplotypic duplication was identified and removed with purge_dups ( Guan *et al.*, 2020). The assembly was scaffolded with Hi-C data ( Rao *et al.*, 2014) using YaHS ( Zhou *et al.*, 2023). The assembly was checked for contamination and corrected as described previously ( Howe *et al.*, 2021). Manual curation was performed using HiGlass ( Kerpedjiev *et al.*, 2018) and Pretext ( Harry, 2022). The mitochondrial genome was assembled using MitoHiFi ( Uliano-Silva *et al.*, 2022), which runs MitoFinder ( Allio *et al.*, 2020) or MITOS ( Bernt *et al.*, 2013) and uses these annotations to select the final mitochondrial contig and to ensure the general quality of the sequence.

A Hi-C map for the final assembly was produced using bwa-mem2 ( Vasimuddin *et al.*, 2019) in the Cooler file format ( Abdennur & Mirny, 2020). To assess the assembly metrics, the *k*-mer completeness and QV consensus quality values were calculated in Merqury ( Rhie *et al.*, 2020). This work was done using Nextflow ( Di Tommaso *et al.*, 2017) DSL2 pipelines “sanger-tol/readmapping” ( Surana *et al.*, 2023a) and “sanger-tol/genomenote” ( Surana *et al.*, 2023b). The genome was analysed within the BlobToolKit environment ( Challis *et al.*, 2020) and BUSCO scores ( Manni *et al.*, 2021; Simão *et al.*, 2015) were calculated.

Table 3 contains a list of relevant software tool versions and sources.

**Table 3.** Software tools: versions and sources.

Software tool	Version	Source
BlobToolKit	4.1.7	https://github.com/blobtoolkit/blobtoolkit
BUSCO	5.3.2	https://gitlab.com/ezlab/busco
Hifiasm	0.16.1-r375	https://github.com/chhylp123/hifiasm
HiGlass	1.11.6	https://github.com/higlass/higlass
Merqury	MerquryFK	https://github.com/thegenemyers/MERQURY.FK
MitoHiFi	2	https://github.com/marcelauliano/MitoHiFi
PretextView	0.2	https://github.com/wtsi-hpag/PretextView
purge_dups	1.2.3	https://github.com/dfguan/purge_dups
sanger-tol/genomenote	v1.0	https://github.com/sanger-tol/genomenote
sanger-tol/readmapping	1.1.0	https://github.com/sanger-tol/readmapping/tree/1.1.0
YaHS	yahs-1.1.91eebc2	https://github.com/c-zhou/yahs

### Wellcome Sanger Institute – Legal and Governance

The materials that have contributed to this genome note have been supplied by a Tree of Life collaborator. The Wellcome Sanger Institute employs a process whereby due diligence is carried out proportionate to the nature of the materials themselves, and the circumstances under which they have been/are to be collected and provided for use. The purpose of this is to address and mitigate any potential legal and/or ethical implications of receipt and use of the materials as part of the research project, and to ensure that in doing so we align with best practice wherever possible. The overarching areas of consideration are:

•   Ethical review of provenance and sourcing of the material

•   Legality of collection, transfer and use (national and international)

Each transfer of samples is undertaken according to a Research Collaboration Agreement or Material Transfer Agreement entered into by the Tree of Life collaborator, Genome Research Limited (operating as the Wellcome Sanger Institute) and in some circumstances other Tree of Life collaborators.

## Data Availability

European Nucleotide Archive: *Shargacucullia verbasci* (mullein moth). Accession number PRJEB56253; https://identifiers.org/ena.embl/PRJEB56253. ( Wellcome Sanger Institute, 2022) The genome sequence is released openly for reuse. The *Shargacucullia verbasci* genome sequencing initiative is part of the Darwin Tree of Life (DToL) project. All raw sequence data and the assembly have been deposited in INSDC databases. The genome will be annotated using available RNA-Seq data and presented through the Ensembl pipeline at the European Bioinformatics Institute. Raw data and assembly accession identifiers are reported in Table 1.

## Author information

Members of the Darwin Tree of Life Barcoding collective are listed here: https://doi.org/10.5281/zenodo.4893703.

Members of the Wellcome Sanger Institute Tree of Life programme are listed here: https://doi.org/10.5281/zenodo.4783585.

Members of Wellcome Sanger Institute Scientific Operations: DNA Pipelines collective are listed here: https://doi.org/10.5281/zenodo.4790455.

Members of the Tree of Life Core Informatics collective are listed here: https://doi.org/10.5281/zenodo.5013541.

Members of the Darwin Tree of Life Consortium are listed here: https://doi.org/10.5281/zenodo.4783558.
