# Supplementary material for: The genome sequence of the Mullein moth, Shargacucullia verbasci (Linnaeus, 1758)
Source: Wellcome Open Res. 2023 Aug 17;8:346. [Version 1] doi: 10.12688/wellcomeopenres.19757.1 (PMC11333875; doi:10.12688/wellcomeopenres.19757.1)
Supplement: Peer review history

# Reviewer report: Reviewer response for version 1

**DOI:** 10.21956/wellcomeopenres.21883.r91420

**Published:** 2024-08-22

**Authors:** Jeffrey Marcus

**Affiliations:** 1 University of Manitoba, Winnipeg, Manitoba, Canada

In this article, the authors describe the sequencing and assembly of the *Shargacucullia verbasci *genome using DNA from a single larval specimen collected in the UK. The primary genome sequence assembly includes proposed chromosomal pseudomolecule sequences for 30 autosomes, the Z sex chromosome, the W sex chromosome and a complete mitochondrial genome. On the basis of the genome assembly, which included a W chromosome, the sex of the specimen was inferred to be female. On the whole, this is a useful contribution to the scientific literature, but please see my comments below, especially regarding the identification of the specimen, the preparation of the specimen for nucleic acid isolation, the role of the RNAseq dataset in the project, and details of mitogenome assembly.

**Some suggestions to the authors:**

1. **Specimen identification:** The individual researcher who did the specimen identification was named, but keys/species descriptions consulted, or the morphological characters used for the identification have not been included in the manuscript. Further, the image included in Figure 1 is not of the specimen that was sequenced, and the Methods appear to describe the removal of the head of the caterpillar, the removal of the gut, and the processing of the remainder of the specimen into “lentil sized species” prior to specimen identification. Morphology-based species identification based on a minced caterpillar body seems problematic.
2. “lentil-sized pieces” I am not a lentil expert, but I will note that dried lentils vary between 3 and 9 mm in length, with geographic variation in human cultural preferences for the size of preferred varieties (larger in Europe and the Middle East, smaller in South and East Asian). Cooked lentils are presumably larger than dried lentils. To which are the authors referring? All of this is to say, as fond of lentils as any of us may be, it would be far more informative for anyone hoping to repeat the nucleic acid protocol if the authors specified the approximate diameter of the pieces using metric measurements.
3. **Sequencing:** Included in this manuscript is a description of the creation of an RNAseq dataset. The authors do not describe how this dataset was used in the genome assembly. If it wasn’t used, it’s not clear why the RNAseq dataset was created or included in this study.
4. The authors describe how “The mitochondrial genome was assembled using MitoHiFi (Uliano-Silva et al. 2022) which runs MitoFinder (Allio et al. 2020) or MITOS (Bernt et al. 2013) and uses these annotations to select the final mitochondrial contig…”. The authors do not describe which of the algorithms generated the selected contig for the mitogenome.

Are sufficient details of methods and materials provided to allow replication by others?

Partly

Is the rationale for creating the dataset(s) clearly described?

Partly

Are the datasets clearly presented in a useable and accessible format?

Yes

Are the protocols appropriate and is the work technically sound?

Yes

Reviewer Expertise:

Evolutionary biology of insects, phylogenomics

I confirm that I have read this submission and believe that I have an appropriate level of expertise to confirm that it is of an acceptable scientific standard, however I have significant reservations, as outlined above.

# Reviewer report: Reviewer response for version 1

**DOI:** 10.21956/wellcomeopenres.21883.r88912

**Published:** 2024-08-19

**Authors:** Andrew G Clark

**Affiliations:** 1 Cornell University, Ithaca, NY, USA

•    Is the work clearly and accurately presented and does it engage with the current literature?

 The paper is a very basic description of the process of generating and assembling the genome sequence of the Mullein moth. It is clearly presented, citing the appropriate papers mostly for software tools to perform the assembly. The sequencing effort was extensive (41x coverage of PacBio HiFi) and represents the state-of-the-art for single-pass de novo assembly. Relatively little hand work was involved in assembling chromosome level scaffolds.

 •    Is the study design appropriate and does the work have academic merit?

 Yes. Everything in the paper is clear and seems accurate. The only thing that diminishes the academic merit is that the authors do not push the analysis very far. It seems odd to produce this sequence and not also present first-pass annotation of the coding genes, transposable elements, and other structural aspects. Lepidoptera are holocentric, and it seems some discussion of the structure of regions that might be involved in chromosome pairing would be interesting. 

 •    Are sufficient details of methods and analysis provided to allow replication by others?

 Generally, the paper is very clear about the procedures (down to use of the bathroom for the dissection).  One detail that was missing was the procedure for isolating large fragment DNA and the distribution of fragment sizes that entered the library preparation.

 •    If applicable, is the statistical analysis and its interpretation appropriate?

 The only statistical analysis was the BUSCO and BLobToolkit analysis of features of the assembly, including contig size, GC content, etc..  Very basic stuff, and there was no interpretation really.  I found the BlobToolkit graphics to be less than satisfying, and simple plots of histograms of contig size, GC content, level of polymorphism seen in comparing the portions with phased haplotypes, etc would have been more informative.

 •    Are all the source data underlying the results available to ensure full reproducibility?

 Yes, all data have been appropriately deposited.

 •    Are the conclusions drawn adequately supported by the results?

 The paper is a very straightforward presentation of a genome assembly project, derived from a single individual moth larva. The sequencing effort was quite extensive (41x coverage of PacBio HiFi) and so the standard tools, like HIFIasm were adequate to produce a quite impressive genome assembly. In short, all the conclusions that are drawn are well supported. It seems strange to have stopped so short of drawing more interesting conclusions, but perhaps the idea is to get the completed genome assembly into the public domain as quickly as possible. 

Are sufficient details of methods and materials provided to allow replication by others?

Yes

Is the rationale for creating the dataset(s) clearly described?

Yes

Are the datasets clearly presented in a useable and accessible format?

Yes

Are the protocols appropriate and is the work technically sound?

Yes

Reviewer Expertise:

Population genetics

I confirm that I have read this submission and believe that I have an appropriate level of expertise to confirm that it is of an acceptable scientific standard.

# Reviewer report: Reviewer response for version 1

**DOI:** 10.21956/wellcomeopenres.21883.r91424

**Published:** 2024-08-12

**Authors:** Alice Dennis

**Affiliations:** 1 University of Namur, Namur, Belgium

**Data Note: **Moth *Shargacucullia verbasci*.

 This report presents the genome assembly of the moth *Shargacucullia verbasci*. This assembly is of high quality and provides a valuable reference for future work. I suggest only some minor edits to clarify the assembly of the two haplotypes, and to remove redundancy

**Minor comments:**

- There is redundancy between the end of "Background" and First sentence under "Genome sequencing report" (these paragraphs follow each other). Some redundancy with beginning of Methods too.
- I assume the numbers in the first line of "Genome sequence report" (52.02, 025) are GPS coordinates, but I would specify this. This is also redundant with the first line of the Methods section, so I would revise to keep just one.
- Second paragraph of "Genome sequencing report". As I understand it, the stats you give are for the first haplotype. Is this correct? It would perhaps make this more clear if you give the stats for the second haplotype as well. And you say both have been deposited. Do they have accessions that can be stated in the text? Were they submitted together?
- On the bottom of page 6 you mention contamination and correction as previously described. Could you please very briefly describe what this entailed. You mention Blobtoolkit further down. Perhaps this belongs up here?
- What data did you give to MitoHifi? The assembly or the raw reads?
- At the end of the methods, you mention BUSCO, reference used. Could you please include these details. Even if they are in the figure it would be nice to have them in the main text too.

Are sufficient details of methods and materials provided to allow replication by others?

Yes

Is the rationale for creating the dataset(s) clearly described?

Yes

Are the datasets clearly presented in a useable and accessible format?

Yes

Are the protocols appropriate and is the work technically sound?

Yes

Reviewer Expertise:

Evolution, ecology and genomics

I confirm that I have read this submission and believe that I have an appropriate level of expertise to confirm that it is of an acceptable scientific standard.
